# Accuracy of large vessel flow evaluation performed by technologists in patients with congenital heart disease using cardiac MRI

**DOI:** 10.1186/1532-429X-15-S1-T7

**Published:** 2013-01-30

**Authors:** Chris Lawton, Robert Bolton, Elisa McAlindon, Mark C Hamilton, Chiara Bucciarelli-Ducci, Nathan Manghat

**Affiliations:** 1 NIHR Cardiovascular Biomedical Research Unit, Bristol, UK

## Background

Adults with complex congenital heart disease, such as Tetralogy of Fallot (ToF) require annual follow up and accurate, detailed physiological evaluation by cardiac magnetic resonance imaging (CMR). This is time consuming for the reporting radiologist with a large proportion of time taken up by multi-vessel flow analysis of the aorta and central pulmonary arteries.

Technologists can significantly contribute to the clinical workflow of a busy cardiac imaging service by performing flow analysis thus reducing radiologist reporting time.

## Methods

The technologists had basic knowledge and a short period of training by a cardiac radiologist in the use of the vessel analysis software.

20 CMR studies of ToF were included in this study. 3 observers performed the analyses: 2 technologists, 1 radiologist. Flow analysis using semi-automated software (Argus, Siemens) of the aorta, main, right and left pulmonary arteries were completed.

10 cases were re-analysed after 24 hours. Results were compared to those of an experienced cardiac radiologist.

## Results

Inter- and intra- observer reproducibility of two variables (Peak velocity, Net forward flow rate; this is derived as a function of antegrade and retrograde flow) was calculated.

1. Interobserver agreement between 1 and 2 (technologists) was excellent (Net flow bias 0.05+/-0.17, Peak velocity bias 0 +/-0.56)

2. Intraobserver agreement for both technologists was excellent (Net flow observer 1 bias 0.03 +/-0.11, observer 2 bias -0.08 +/- 0.13; peak flow observer 1 bias 0.5 +/-1.26, observer 2 bias 0.4 +/-1.26)

3. Interobserver agreement between technologist and radiologist very good for net flow (bias 0.06 +/- 0.34), however lower for peak velocity (bias 3.3 +/- 11.4)

## Conclusions

The results demonstrate an overall high level of agreement between the technologist and radiologist flow analysis. This pilot study would suggest that the technologist can facilitate CMR workflow for net flow measurements. However, the discrepancy for technologist vs radiologist peak velocity measurements may be an ‘experience effect' of inclusion of artefact/ over-contouring and whilst this will require further training before implementation in clinical practice, the observed differences are unlikely to have a significant clinical implication.

## Funding

Biomedical Research Unit. NIHR

**Figure 1:**
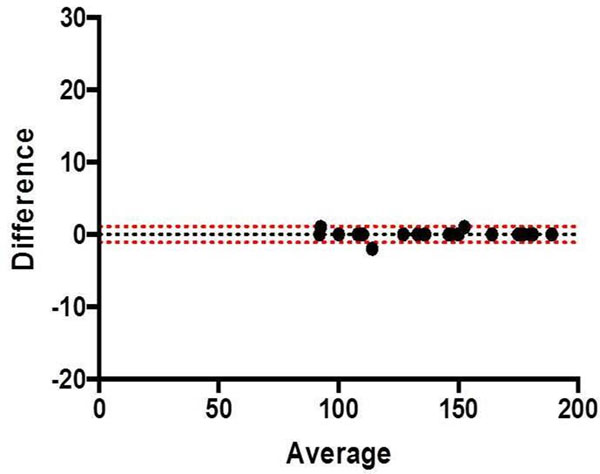
Bland-Altman: Inter Observer 1 vs 2 Peak Velocity (cm/s)

**Figure 2:**
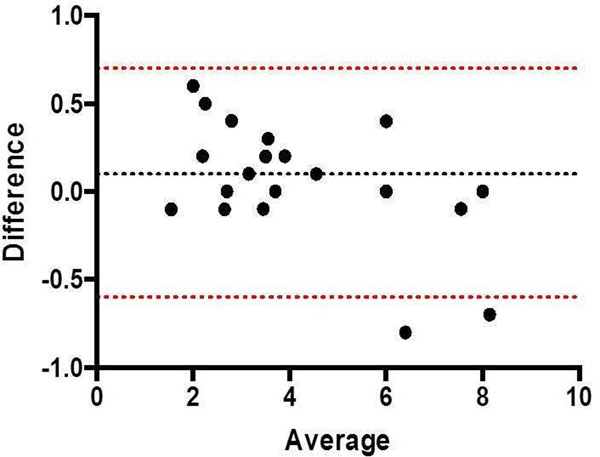
Bland-Altman: Inter Observer 1 vs 3 Net Flow (l/min)

